# Essential and distinct roles of the F-box and helicase domains of Fbh1 in DNA damage repair

**DOI:** 10.1186/1471-2199-9-27

**Published:** 2008-03-03

**Authors:** Chikako Sakaguchi, Takashi Morishita, Hideo Shinagawa, Takashi Hishida

**Affiliations:** 1 Laboratory of Genome Dynamics, Research Institute for Microbial Diseases, Osaka University, Osaka 565-0871, Japan; 2 BioAcademia Inc., Ibaraki, Osaka 565-0085, Japan

## Abstract

**Background:**

DNA double-strand breaks (DSBs) are induced by exogenous insults such as ionizing radiation and chemical exposure, and they can also arise as a consequence of stalled or collapsed DNA replication forks. Failure to repair DSBs can lead to genomic instability or cell death and cancer in higher eukaryotes. The *Schizosaccharomyces pombe fbh1 *gene encodes an F-box DNA helicase previously described to play a role in the Rhp51 (an orthologue of *S. cerevisiae RAD51*)-dependent recombinational repair of DSBs. Fbh1 fused to GFP localizes to discrete nuclear foci following DNA damage.

**Results:**

To determine the functional roles of the highly conserved F-box and helicase domains, we have characterized *fbh1 *mutants carrying specific mutations in these domains. We show that the F-box mutation *fbh1-fb *disturbs the nuclear localization of Fbh1, conferring an *fbh1 *null-like phenotype. Moreover, nuclear foci do not form in *fbh1-fb *cells with DNA damage even if Fbh1-fb is targeted to the nucleus by fusion to a nuclear localization signal sequence. In contrast, the helicase mutation *fbh1-hl *causes the accumulation of Fbh1 foci irrespective of the presence of DNA damage and confers damage sensitivity greater than that conferred by the null allele. Additional mutation of the F-box alleviates the hypermorphic phenotype of the *fbh1-hl *mutant.

**Conclusion:**

These results suggest that the F-box and DNA helicase domains play indispensable but distinct roles in Fbh1 function. Assembly of the SCF^Fbh1 ^complex is required for both the nuclear localization and DNA damage-induced focus formation of Fbh1 and is therefore prerequisite for the Fbh1 recombination function.

## Background

Homologous recombination (HR) is a major error-free pathway of DSB repair found in all organisms thus far examined (for reviews, see [1-3]). Extensive studies of HR repair mechanisms in the budding yeast *Saccharomyces cerevisiae *have shown that HR requires members of the *RAD52 *epistasis group, including *RAD50*, *MRE11*, *XRS2*, *RAD51*, *RAD54*, *RAD55*, *RAD57*, and *RAD59 *[4-6]. More recent studies of HR mechanisms in the fission yeast *Schizosaccharomyces pombe *have revealed many similarities with HR in *S. cerevisiae *and have led to many insights into the mechanisms of HR-dependent DSB repair and the identification of novel genes with homologues in higher eukaryotes [7,8]. For example, Rhp51-interacting proteins such as the Swi5-Sfr1 mediator complex function in a separate pathway from Rhp55-Rhp57 to promote an Rhp51 strand exchange reaction [9,10]. Furthermore, the *fbh1 *gene encodes a protein consisting of a unique domain architecture, with N-terminal F-box and C-terminal DNA helicase domains [11,12], which is conserved in mammals but not in *S. cerevisiae*. The Fbh1 protein was originally identified as a 3' to 5' DNA helicase that is stimulated by RPA at low ATP concentrations [13]. The helicase domain of Fbh1 is structurally related to the Rep, UvrD, PcrA, and Srs2 family of helicases [14]. Previous studies have shown that *S. cerevisiae *Srs2 regulates *RAD52*-dependent HR by actively disrupting the Rad51 nucleoprotein filament [15,16]. Interestingly, in contrast to Fbh1, Srs2 is conserved in budding and fission yeasts but not in mammals. In *S. pombe*, the *fbh1*Δ mutation is lethal when combined with the *srs2*Δ mutation, and this synthetic lethality can be suppressed by a loss of HR functions [11,12]. Recently, *Chiolo et al*. reported that human FBH1 (hFBH1) suppresses specific recombination defects of *S. cerevisiae srs2 *mutants and that the F-box domain is essential for hFBH1 functions in this respect [17]. Thus, the Fbh1 and Srs2 helicases appear to have only partially analogous functions in controlling HR after the formation of Rhp51 nucleoprotein filaments.

F-box proteins were first characterized as components of SCF ubiquitin-ligase complexes containing Skp1, Cullin, and F-box proteins, in which they bind substrates for ubiquitin-mediated proteolysis [18-21]. The F-box motif consists of 40–50 amino acids and is required for binding to SKP1. Therefore, the F-box motif links F-box proteins to other components of the SCF complex. Indeed, hFBH1 was shown to form an SCF complex and to have ubiquitin ligase activity *in vitro *[14,22]. However, the physiological substrates of SCF^Fbh1 ^are still unknown.

In this study, we characterize the *in vivo *function of Fbh1, focusing on the role of its F-box and helicase domains. Our results demonstrate that the F-box domain of Fbh1 is required for its recruitment to the nucleus and to DNA damage sites, whereas the helicase domain is involved in DNA processing after the Rhp51-dependent step of HR. Thus, both domains have indispensable but distinct roles in Fbh1 functions, and assembly of the SCF^Fbh1 ^complex is a prerequisite for its DNA recombination activities.

## Results and Discussion

### Construction of *fbh1 *mutants with substitutions in the F-box or helicase motif

**Figure 1:**
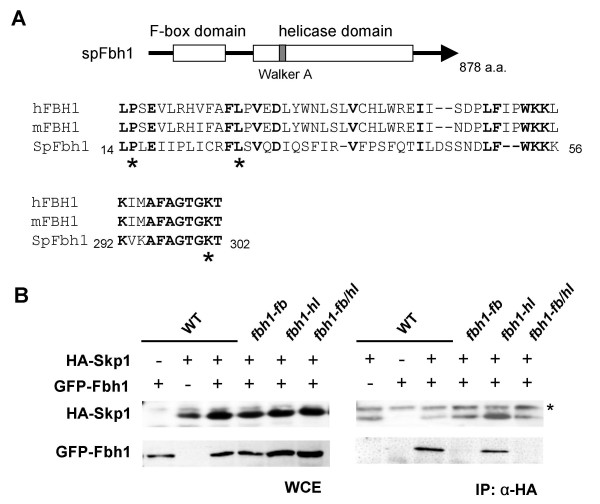
**Construction of *fbh1 *mutants with substitutions in the F-box or helicase motif**. (A) Schematic structure of the Fbh1 protein with the F-box and helicase domains. Sequence alignments of F-box (upper panel) and Walker A (bottom panel) motifs of *S. pombe *(SpFbh1), mouse (mFbh1), and human Fbh1 (hFbh1). Identical amino acids are shown in bold. Asterisks show the positions of the P15A/L26A and K301A mutations. (B) Co-immunoprecipitation of Skp1 with Fbh1. Cells expressing HA-Skp1 and GFP-Fbh1 were cultured as described in the Methods, and cellular extracts were immunoprecipitated with anti-HA antibodies. Samples were resolved by SDS-PAGE followed by western blotting with anti-GFP or anti-HA antibodies. *, non-specific bands.

Fbh1 has a highly conserved N-terminal F-box motif and seven C-terminal helicase motifs. To gain insights into the roles of the F-box and helicase motifs in DNA repair, we constructed two *fbh1 *mutants, *fbh1-fb *and *fbh1-hl*, in which alanine replaces the Pro^15 ^and Leu^26 ^residues within the F-box motif and the Lys^301 ^residue within the Walker A motif of the helicase domain, respectively (Fig. 1A). The highly conserved Pro^15 ^and Leu^26 ^residues in the F-box motif are essential for binding to Skp1 [21,23-25], and Lys^301 ^is a conserved catalytic residue in the Walker A motif essential for ATPase activity [26]. To determine whether the *fbh1-fb *mutant is defective in binding to Skp1, we performed a co-immunoprecipitation assay using HA-tagged Skp1 and GFP-tagged versions of Fbh1. GFP-fused *fbh1*, *fbh1-fb*, *fbh1-hl*, or *fbh1-fb/hl *alleles were integrated into the genome at the *ars1 *locus in an *fbh1*Δ strain and expressed under the control of the *nmt1 *promoter. GFP-Fbh1 complemented the repair deficiency of the *fbh1 *deletion strain (data not shown) [11], indicating that GFP-Fbh1 and Fbh1 function similarly *in vivo*. We found that wild type Fbh1 and Fbh1-hl, but not Fbh1-fb, co-immunoprecipitated with HA-tagged Skp1 (Fig. 1B), indicating that the *fbh1-fb *mutation prevents association with SCF components.

### The role of the F-box domain

**Figure 2:**
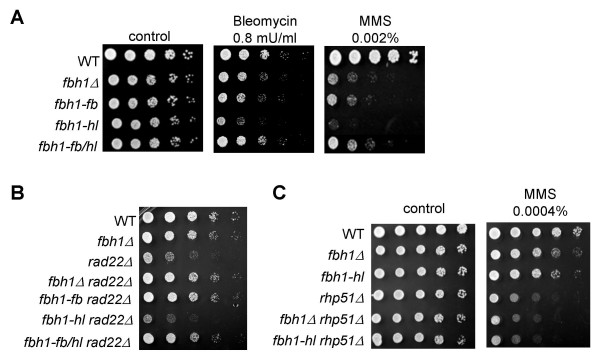
**F-box and helicase mutants are deficient in DNA damage repair**. (A) Wild type and *fbh1*Δ, *fbh1-fb, fbh1-hl*, and *fbh1-fb/hl *mutant cells were grown in liquid YES. Cells were diluted and spotted onto YES plates with the indicated DNA damaging agents as described in the Methods. The plates were incubated for 3 days at 30°C. (B) Cells were grown and spotted onto YES plates. The plates were incubated for 4 days at 30°C. (C) Cells were grown and spotted onto YES plates containing the indicated concentrations of MMS. The plates were incubated for 3 days at 30°C.

The *fbh1*Δ mutation confers hypersensitivity to DNA damaging agents and suppresses the slow growth of a *rad22*Δ strain, which is defective in an orthologue of *S. cerevisiae RAD52 *[11,12]. To examine the effect of the *fbh1 *mutations on these phenotypes, they were introduced into the *S. pombe *genome at the endogenous *fbh1 *locus. The *fbh1-fb *mutation conferred methyl methanesulfonate (MMS) and bleomycin sensitivities similar to those of the *fbh1*Δ mutant, and it suppressed the poor growth phenotype of the *rad22*Δ strain to a similar extent as the *fbh1*Δ mutation (Fig. 2A and 2B). Thus, the *fbh1-fb *mutant is defective in binding to Skp1, and the *fbh1-fb *mutation behaves like the *fbh1*Δ mutation with respect to DNA damage sensitivity and the suppression of the poor growth of *rad22*Δ cells. These results are consistent with a recent study showing that hFBH1 suppresses the hypersensitivity of *S. cerevisiae srs2*Δ cells to DNA damaging agents and that the F-box domain of hFBH1 is essential for this effect [17]. In another study, Osman et al. showed that the F-box domain plays a minor role in Fbh1 function because an F-box mutant (L14A/P15A) created in a previous study has no or little sensitivity to DNA damaging agents [12]. This discrepancy may arise from the use of different F-box mutants. Our F-box mutant (P15A/L26A) is completely defective in binding to Skp1, while in the previous study, the *fbh1 *L14A/P15A mutant had not been characterized in this regard. We suppose that the *fbh1 *L14A/P15A mutation may not fully inactivate the F-box, as previously discussed [12].

### The role of the DNA helicase domain

We next examined the effect of the *fbh1-hl *mutation on Fbh1 function. The *fbh1-hl *mutant shows greater sensitivity to DNA damaging agents than does the *fbh1*Δ mutant (Fig. 2A). In addition, ectopic expression of the *fbh1-hl *allele renders wild type cells sensitive to MMS, suggesting that the *fbh1-hl *alteration is a dominant mutation (see Additional file 1). Previous studies suggest that Fbh1 promotes HR repair by controlling the action of Rhp51 [11,12], which contributes to the suppression of inappropriate recombination events. Therefore, one possibility to explain the *fbh1-hl*-dependent toxic phenotype is that toxic recombination intermediates caused by faulty Rhp51-dependent HR accumulate to a greater extent in the *fbh1-hl *mutant than in the *fbh1*Δ mutant. To test this possibility, we examined the MMS sensitivity of the *fbh1-hl *strain in an *rhp51*Δ background. As expected, *fbh1-hl *cells were as sensitive to MMS as *fbh1*Δ cells with the *rhp51*Δ background (Fig. 2C). These results indicate that Fbh1-hl is not only defective in Fbh1 function but also that it interferes with Rhp51-dependent HR. In addition, the *fbh1-hl *mutation does not suppress the poor growth of *rad22*Δ cells, but rather, it exacerbates their decreased growth rate (Fig. 2B). However, the *fbh1-fb/hl *mutant, which has alterations within both the F-box and Walker A motifs, is as sensitive to DNA damaging agents as the *fbh1-fb *or *fbh1*Δ strains (Fig. 2A), and mutations affecting both domains suppress the slow growth phenotype of the *rad22*Δ strain (Fig. 2B), suggesting that F-box activity is likely to be a prerequisite for helicase activity. Thus, the F-box and DNA helicase domains play indispensable but distinct roles in Fbh1 function.

### Focus formation of *fbh1 *mutants in response to DNA damage

**Figure 3:**
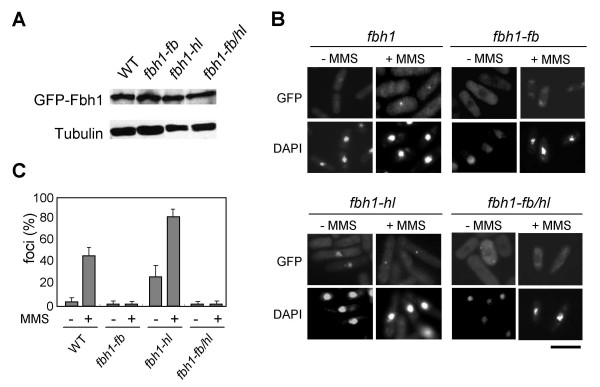
**DNA damage-induced focus formation of Fbh1 mutant proteins**. (A) Protein extracts were prepared from the indicated strains expressing GFP-wild type or mutant *fbh1*. Samples were analyzed by SDS-PAGE followed by western blotting with anti-GFP or anti-α-tubulin antibodies. (B) Cells expressing GFP-wild type or mutant *fbh1 *were incubated in EMM2 without or with MMS (0.1%) for 2 h at 30°C and observed by fluorescence microscopy. The upper and lower panels show GFP and DAPI images, respectively. The scale bar indicates 10 μm. (C) Quantitative analysis of GFP-Fbh1 foci. Cells with Fbh1 foci were counted and divided by the total number of cells. More than 150 individual cells were scored for each strain. The result represents the average of three independent measurements.

Since GFP-Fbh1 is predominantly detected in the nucleus and forms foci in response to DNA damage [11], we assessed DNA damage-induced focus formation in the *fbh1 *mutants. Exponentially growing cells were incubated in EMM2 medium containing 0.1% MMS in the absence of thiamine for 2 h, and GFP-Fbh1 was localized by fluorescence microscopy. The levels of expression of the GFP-fused wild type Fbh1 and of the three mutant Fbh1 proteins were comparable (Fig. 3A). Five percent of untreated cells expressing wild type GFP-Fbh1 contained foci, and 47% of these cells contained foci following MMS treatment (Fig. 3B and 3C). Interestingly, no foci were visible in cells expressing GFP-Fbh1-fb, even after exposure to MMS (Fig. 3B and 3C), indicating that the F-box domain is required for Fbh1 focus formation. In contrast, 27% of untreated cells expressing GFP-Fbh1-hl had foci, and this percentage was dramatically larger than that of cells expressing wild type GFP-Fbh1 (Fig. 3B and 3C). Following MMS treatment, the proportion of cells with foci further increased to 78% (Figure 3B and 3C). One possible explanation for the increased focus formation of *fbh1-hl *cells is that Fbh1-hl can localize to DNA damage sites but not complete DNA processing because it lacks helicase activity, leading to its accumulation at these sites and interference with the HR pathway. Moreover, the additional presence of the F-box mutation almost abolished focus formation in Fbh1-hl cells irrespective of the presence of DNA damage (Fig. 3B and 3C). Taken together, these results suggest that the F-box domain is required for the recruitment of Fbh1 to DNA damage sites and that the helicase domain is required to mediate the HR process.

### Fbh1 is required for the DNA damage-induced formation of Skp1 nuclear foci

**Figure 4:**
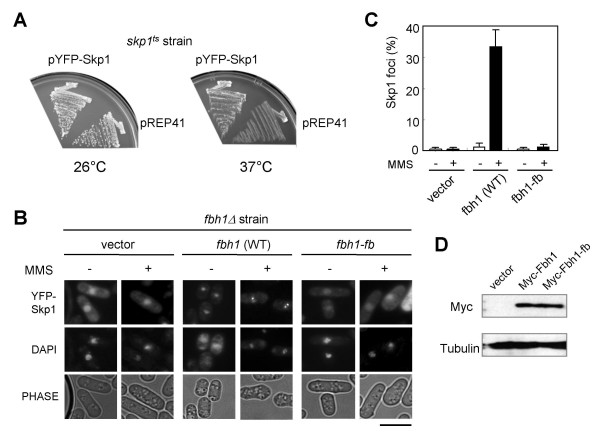
**Skp1 forms DNA-damage-induced foci in an Fbh1-dependent manner**. (A) YFP-Skp1 complements the *skp1*^*ts *^mutants. The *skp1*^*ts *^mutants expressing YFP-wild type *skp1 *or none in the absence of thiamine were streaked onto EMM2 plates and incubated at 26°C (left) or 37°C (right) for 3 days. (B) DNA damage-induced YFP-Skp1 foci. The *fbh1*Δ strains harbouring the vector pREP41, pREP42 Myc-*fbh1 *or pREP42 Myc-*fbh1-fb *were transformed with pREP42 YFP-*skp1*. Cells were treated with or without MMS (0.1%) for 2 h at 30°C and observed by fluorescence microscopy. The top, middle, and bottom panels show YFP, DAPI, and phase contrast images, respectively. The scale bar indicates 10 μm. (C) Quantitative analysis of YFP-Skp1 foci. Cells with Skp1 foci were counted and divided by the total number of cells. At least 100 cells were counted per strain. The result represents the average of three independent measurements. (D) Expression of Myc-tagged Fbh1. Cells were treated as in (B) and protein extracts were analysed by SDS-PAGE followed by Western blotting with anti-Myc or anti-α-tubulin antibodies.

Since Fbh1 is assembled into the SCF complex, we next examined the subcellular localization of Skp1 by fusing YFP to its N terminus. The resulting fusion protein was expressed from a plasmid under the control of the *nmt1 *promoter. YFP-Skp1 functions normally *in vivo*, since it fully complements the temperature sensitivity of an *skp1*^*ts *^mutant (Fig. 4A). When the YFP-Skp1 was expressed in *fbh1*Δ cells expressing wild-type *fbh1 *or *fbh1-fb*, it was detected in both the nucleus and cytoplasm, with a higher level of the protein in the nucleus. YFP-Skp1 foci were not detected in *fbh1*Δ cells expressing wild type *fbh1*, but following 1 h exposure to MMS, most of the cells had nuclear foci (Fig. 4B and 4C). Remarkably, *fbh1*Δ cells expressing the *fbh1-fb *mutant did not have any YFP-Skp1 foci, even in the presence of MMS (Fig. 4B and 4C). Wild-type *fbh1 *and *fbh1-fb *were expressed at a similar level (Fig. 4D). Thus, these results indicate that Skp1 focus formation is dependent on the F-box domain of Fbh1.

### The F-box domain is responsible for the nuclear localization of Fbh1

**Figure 5:**
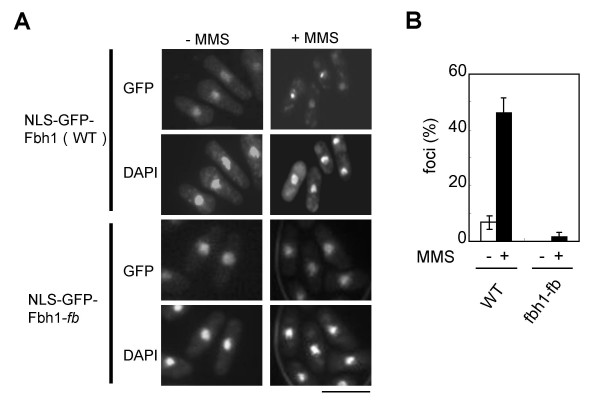
**The F-box motif is required for DNA damage-induced focus formation of Fbh1**. (A) *fbh1*Δ cells expressing NLS-GFP-Fbh1 (wild type) and NLS-GFP-Fbh1-fb were treated as in Figure 3 with MMS (0.1%) for 2 h at 30°C and observed by fluorescence microscopy. The upper and lower panels show GFP and DAPI images, respectively. The scale bar indicates 10 μm. (B) Quantitative analysis of NLS-GFP-Fbh1 foci. Cells with Fbh1 foci were counted and divided by the total number of cells. At least 100 cells were counted per strain. The result represents the average of three independent measurements.

In the course of our studies, we noticed that the *fbh1-fb *mutation affected the subcellular localization of Fbh1. As shown in Fig. 3B, Fbh1-fb and Fbh1-fb/hl showed predominantly cytoplasmic localization and little GFP signal was seen in the nucleus, in striking contrast with the subcellular localization of wild type Fbh1. These results indicate that the *fbh1-fb *mutation disturbs the nuclear localization of Fbh1. Although this interpretation could explain why the *fbh1-fb *mutation confers an *fbh1 *null phenotype and suppresses the toxic phenotypes of the *fbh1-hl *mutation, it is still unknown whether SCF^Fbh1 ^complex formation is required for DNA damage-induced nuclear focus formation. To test this possibility, a NLS sequence from the simian virus 40 large-T antigen (PKKKRKV) [27] was fused to the GFP-Fbh1 and GFP-Fbh1-fb constructs at their N termini, and focus formation in response to DNA damage was examined. As with GFP-Fbh1 cells, these constructs were integrated into the genome at the *ars1 *locus in the *fbh1*Δ strain. Control experiments showed that NLS-GFP-Fbh1 but not NLS-GFP-Fbh1-fb complemented the MMS sensitivity of the *fbh1*Δ strain (data not shown). As expected, NLS-GFP-Fbh1 formed discrete foci in cells exposed to MMS (Fig. 5). NLS-GFP-Fbh1-fb was also detected in the nucleus like wild type Fbh1, but it still failed to form foci in response to DNA damage (Fig. 5). These results suggest that the F-box domain is required for the nuclear localization and DNA damage-induced focus formation of Fbh1. It should be noted that the GFP-F-box domain mutant could not enter the nucleus (see Additional file 2), suggesting that in addition to the F-box domain, another domain of Fbh1 might also be important for its nuclear targeting.

An important unresolved issue is the identification of physiological substrates of SCF^Fbh1 ^that presumably regulate the HR pathway. A recent study has shown that hFBH1 has a short half-life when it is expressed in *S. cerevisiae*, and its degradation depends on the presence of a functional F-box and yeast SCF components [17], suggesting that one candidate for a SCF^hFBH1 ^substrate is hFBH1 itself. The rapid turnover of hFBH1 might contribute to the tight regulation of hFBH1 helicase activity. However, if SCF^hFBH1 ^complex formation is also necessary for its nuclear transport and recruitment to DNA damage sites, the alteration in subcellular localization caused by the F-box mutation might affect hFBH1 stability in budding yeast cells. In addition, many F-box proteins identified to date target more than one substrate for degradation [28-30]. Since our present data suggest that Fbh1 functions as an SCF^Fbh1 ^ubiquitin ligase complex for HR repair, the most plausible candidates as SCF^Fbh1 ^substrates are HR proteins, including Rhp51. For example, SCF^Fbh1 ^might contribute to regulate Rhp51-dependent HR by promoting the ubiquitination of Rhp51 or other recombination proteins. Future studies will be needed to determine whether Fbh1 is a physiological SCF^Fbh1 ^substrate and to identify other SCF^Fbh1 ^substrates, which would provide a means to conduct a more detailed analysis of its function in HR repair.

## Conclusion

In this study, we characterized the *in vivo *function of Fbh1, focusing on the role of its F-box and helicase domains. Our results show that the assembly of SCF^Fbh1 ^mediated by the F-box domain of Fbh1 is required for its recruitment to both the nucleus and DNA damage sites, whereas the helicase domain is involved in controlling the action of Rhp51. Thus, Fbh1 is tightly regulated by SCF components, which might prevent it from functioning inappropriately in HR.

## Methods

### *S. pombe *strains and plasmids

All yeast strains used in this study are listed in Table 1. *S. pombe *cells were grown in YES or EMM medium, and standard genetic and molecular procedures were employed as previously described [31]. An *fbh1 *cDNA clone was constructed by PCR from total *S. pombe *cDNA with the primers CSF (5'-GGCGGATCCCATATGAGTGCTCAACATTTACA-3') and CSR (5'-GGCGGATCCCTACTGATCATGTACAGCAAA-3'). The *fbh1 *cDNA fragment was cloned into the *Bam*HI site of pUC119 to produce pUCcfbh1. An *fbh1 *genomic DNA was obtained from a genomic library [11], and a *Bam*HI-*Kpn*I fragment containing the *fbh1 *coding region was cloned into pUC119 to produce pUCgfbh1. The *fbh1-fb *and *fbh1-hl *mutant genes were constructed by PCR-mediated site-directed mutagenesis of pUCcfbh1 and pUCgfbh1. All mutant clones were sequenced to ensure that only the desired mutation had been introduced. *Bam*HI-*Kpn*I fragments of the *fbh1 *mutants were introduced into the vector pU19, which carries the *ura4*^+ ^gene for directing gene replacement. The resulting plasmids were digested with *Age*I and integrated into the *S. pombe *genome. Transformed strains were then plated onto EMM2 plates containing 5-fluoroorotic acid to select *ura- *cells.

**Table 1:** Strains used in this study

**Strain**	**Genotype**	**Source**
MP111	*h*^+ ^*leu1-32 ura4-D18*	[11]
MPF1	*h*^+ ^*fbh1*::*LEU2 leu1-32 ura4-D18*	[11]
12521	*h*^+ ^*fbh1*::*KanMX-leu1-32 ura4-D18 ade6-704*	Lab. stock
C11	*h*^+^*leu1-32 ura4-D18 fbh1-fb*	This study
C12	*h*^+ ^*leu1-32 ura4-D18 fbh1-hl*	This study
C13	*h*^+^*leu1-32 ura4-D18 fbh1-fb/hl*	This study
C100	*h*^+ ^*ars1*::pREP41-EGFP N-Fbh1 *leu1-32 ura4-D18 ade6-704 fbh1::KanMX*	This study
C101	*h*^+^*ars1*::pREP41-EGFP N-Fbh1-*fb leu1-32 ura4-D18 ade6-704 fbh1::KanMX*	This study
C102	*h*^+ ^*ars1*::pREP41-EGFP N-Fbh1-*hl leu1-32 ura4-D18 ade6-704 fbh1::KanMX*	This study
C103	*h*^+^*ars1*::pREP41-EGFP N-Fbh1-*fb/hl leu1-32 ura4-D18 ade6-704 fbh1::KanMX*	This study
B54	*smt-0 rhp51::his3*^+^*leu1-32 ura4-D18 his3-D1 arg3-1*	Y. Tsutsui
C109	*rhp51::his3*^+^*fbh1-hl leu1-32 ura4-D18 his3-D1 arg3-1*	This study
C15	*smt-0 rad22::arg3*^+^*ura4-D18 leu1-32 arg3-D1*	Lab. stock
C105	*mat1PD::LEU2 rad22::arg3*^+^*fbh1::KanMX ura4-D18 leu1-32 arg3-D1*	This study
C106	*smt-0 rad22::arg3*^+^*fbh1-fb ura4-D18 leu1-32 arg3-D1*	This study
C107	*mat1PD::LEU2 rad22::arg3*^+^*fbh1-hl ura4-D18 leu1-32 arg3-D1*	This study
C108	*smt-0 rad22::arg3*^+^*fbh1-fb/hl leu1-32 ura4-D18 his3-D1 arg3-1*	This study
	*skp1-94 ura4-D18 leu1-32 his3-D1*	T. Toda
MPF25	*smt-0 fbh1::LEU2 rhp51::his3*^+^*ura4-D18 leu1-32 his3-D1 arg3-D1*	[11]

### Expression of the GFP-Fbh1 and YFP-Skp1 fusions in *S. pombe*

Wild type and mutant *fbh1 *cDNAs were cloned separately into the vector pREP41 EGFP N [32] to express enhanced green fluorescent protein (EGFP) fusion proteins under the control of the medium-strength *nmt1 *promoter. The resulting plasmids were linearized at the unique *Mlu*I site within the *ars1 *sequence of the plasmid pREP41 and then introduced into the *ars1 *locus of the *fbh1*Δ strain. To construct pREP41/NLS-GFP-Fbh1-fb, two complementary DNA oligonucleotides encoding a NLS sequence (PKKKRKV) from the SV40 large T antigen were synthesized and inserted at the N-terminus-encoding region. The *skp1 *cDNA was cloned into the plasmid pREP41 YFP N to express a yellow fluorescent protein-Skp1 fusion under the control of the *nmt1 *promoter. Cells harboring pREP41 YFP-Skp1 were grown in EMM2 medium with appropriate supplements and containing 0.1% MMS in the absence of thiamine for 2 h. Cells were fixed with 70% ethanol and observed by fluorescence microscopy. More than 100 individual cells were scored for each strain.

### Spot assays

Logarithmically growing cells were harvested and resuspended in water. Five-fold serial dilutions of cultures of the indicated mutants were spotted onto YES plates containing the indicated concentration of chemical genotoxins. Plates were incubated at 30°C for 3–4 days. All spot assays were repeated at least twice to ensure that the results were reproducible.

### Immunoprecipitation

The *skp1 *fragment was amplified by PCR from an *S. pombe *cDNA library and cloned into the vector pREP42 HA N, which encodes a triple C-terminal hemagglutinin (HA) tag [32]. The *fbh1 *mutant strains C100, C101, C102 and C103 were transformed with pREP42 HA N or pREP42 HA-Skp1. The transformants were grown in EMM2 medium with appropriate supplements in the absence of thiamine to express N-terminally HA-tagged *skp1 *from the thiamine-repressible *nmt1 *promoter. Mid-log-phase cells from a 50-ml culture were collected, washed with buffer A (50 mM Tris-HCl, pH 7.5, 15 mM EGTA, 100 mM NaCl, 0.1% (w/v) Triton ×-100, protease inhibitor cocktail for yeast (Sigma), 1 mM dithiothreitol, 1 mM phenylmethylsulfonyl fluoride) and resuspended in 500 μl buffer A. Cells were disrupted with the same volume of acid-washed glass beads using a Mini-BeadBeater-8 (BioSpec Products). The supernatant fraction was collected by centrifugation and used for immunoprecipitation. Fifty microliters of protein G-agarose (GE Healthcare) was added to absorb nonspecific Protein G binding protein. Twenty microliters of anti-HA antibody (12CA5, Roche) and 40 μl Protein G-agarose were used per 400 μl cell lysate, and the mixture was rotated for 2 h at 4°C. The beads were washed three times with buffer A, resuspended in 25 μl 5% SDS-polyacrylamide gel electrophoresis (PAGE) sample buffer, and boiled for 5 min. After centrifugation, the supernatants were separated by SDS-PAGE and analyzed by western blotting with an ECL Advance Western blotting detection kit (GE Healthcare).

### Preparation of yeast extracts and Western Blotting

Total protein extract was prepared from 5 × 10^6 ^cells from logarithmically growing culture as described previously [11]. Proteins were analyzed by SDS-PAGE, transferred to PVDF membranes, and probed with anti-Myc monoclonal antibody (Roche) or anti-a-tubulin antibody (Sigma). Detection was performed with HRP-conjugated secondary antibodies followed by treatment using the ECL advance Western blot detection kit (GE Healthcare).

## Authors' contributions

CS performed experiments and drafted the manuscript. TM and HS participated in the experimental design and analyzed the data. TH performed experiments, participated in the experimental design, analyzed the data and finalized the manuscript. All authors read and approved the final manuscript.

## Supplementary Material

Additional file 1 **The *fbh1-fb *allele is dominant for DNA repair**. Wild type cells expressing GFP-wild type or mutant *fbh1 *under the control of the *nmt1 *promoter at the *ars1 *locus were incubated in EMM2 and spotted onto EMM2 plates containing MMS. The plates were incubated for 3 days at 30°C. Click here for file

Additional file 2 **GFP-F-box domain mutant could not enter the nucleus**. *fbh1*Δ cells expressing GFP-F-box (1–269 amino acids) were incubated in EMM2 without or with MMS (0.1%) treatment for 2 h at 30°C and observed by fluorescence microscopy. The upper and lower panels show GFP and DAPI images, respectively. The scale bar indicates 10 μm. Click here for file
