# Supplementary figures and images for: Essential and distinct roles of the F-box and helicase domains of Fbh1 in DNA damage repair
Source: BMC Mol Biol. 2008 Mar 3;9:27. doi: 10.1186/1471-2199-9-27 (PMC2294136; doi:10.1186/1471-2199-9-27)

## WT strain

---

Control

MMS 0.001%

GFP fbh1 (WT)

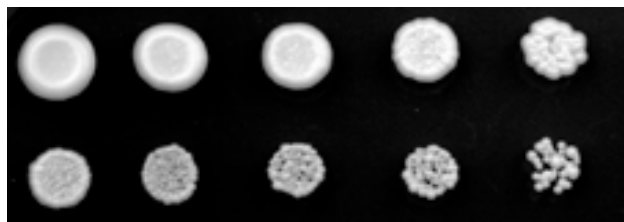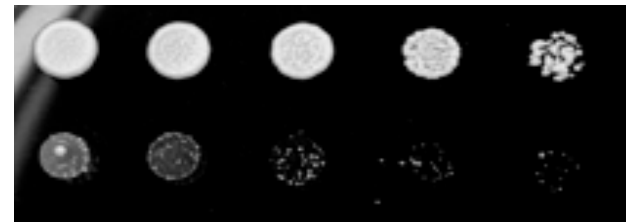

GFP fbh1-*hl*

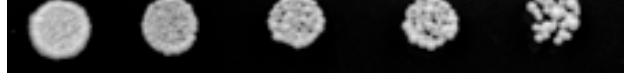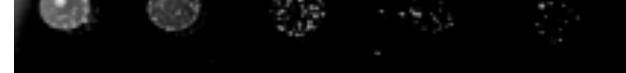

GFP fbh1 (WT)

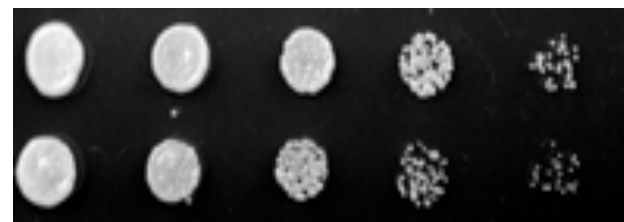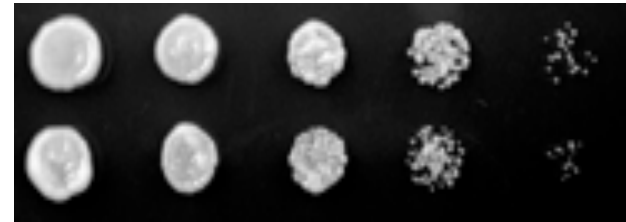

GFP fbh1-*fb*

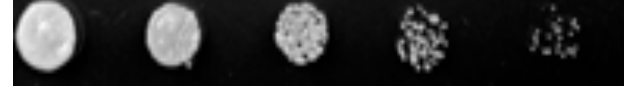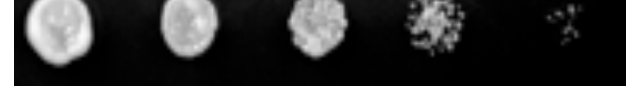

Supplement: Additional file 1 — The fbh1-fb allele is dominant for DNA repair. Wild type cells expressing GFP-wild type or mutant fbh1 under the control of the nmt1 promoter at the ars1 locus were incubated in EMM2 and spotted onto EMM2 plates containing MMS. The plates were incubated for 3 days at 30°C. [file 1471-2199-9-27-S1.pdf]

## GFP-F-box

---

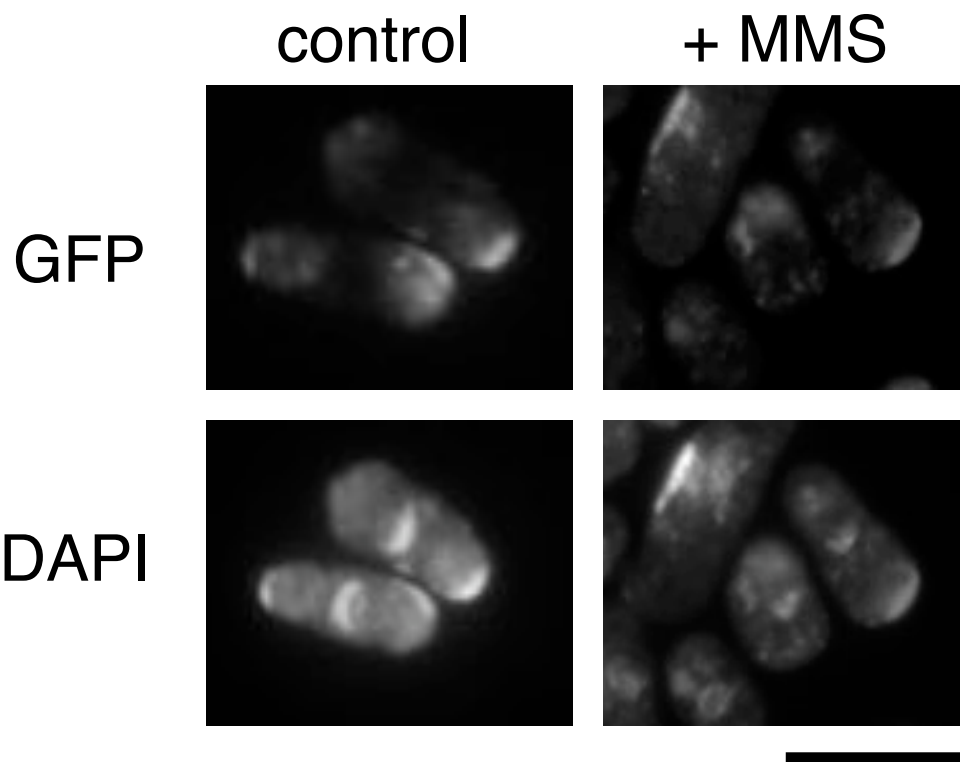

Supplement: Additional file 2 — GFP-F-box domain mutant could not enter the nucleus. fbh1Δ cells expressing GFP-F-box (1–269 amino acids) were incubated in EMM2 without or with MMS (0.1%) treatment for 2 h at 30°C and observed by fluorescence microscopy. The upper and lower panels show GFP and DAPI images, respectively. The scale bar indicates 10 μm. [file 1471-2199-9-27-S2.pdf]
